# Containment of COVID-19 outbreaks with lower incidence and case fatality rates in long-term care facilities by early intervention of emergency response teams

**DOI:** 10.1371/journal.pone.0287675

**Published:** 2023-06-27

**Authors:** Kouki Akahoshi, Keiko Nakamura, Hisayoshi Kondo, Akinori Wakai, Yuichi Koido

**Affiliations:** 1 Department of Global Health Entrepreneurship Division of Public Health Tokyo Medical and Dental University (TMDU), Bunkyo, Tokyo, Japan; 2 DMAT Secretariat, National Hospital Organization Headquarters, Tachikawa, Tokyo, Japan; Bach Mai Hospital, VIET NAM

## Abstract

**Objective:**

To examine whether post-outbreak early-phase interventions by emergency response teams (ERTs) in long-term care facilities (LTCFs) contribute to containment with lower incidence and case-fatality rate of COVID-19 and analyse the required assistance.

**Methods:**

Records from 59 LTCFs (28 hospitals, 15 nursing homes, and 16 homes) assisted by ERTs after the COVID-19 outbreak, between May 2020 and January 2021, were used for the analysis. Incidence and case-fatality rates among 6,432 residents and 8,586 care workers were calculated. The daily reports of ERTs were reviewed, and content analysis was performed.

**Results:**

Incidence rates among residents and care workers with early phase (<7 days from onset) interventions (30·3%, 10·8%) were lower than those with late phase (≥7 days from onset) interventions (36·6%, 12·6%) (p<0·001, p = 0·011, respectively). The case-fatality rate among residents with early-phase and late-phase interventions were 14·8% and 16·9%, respectively. ERT assistance in LTCFs was not limited to infection control but extended to command and coordination assistance in all studied facilities.

**Conclusion:**

Assistance in the facility’s operational governance from the early phase of an outbreak in LTCFs contributed to a significant decline in incidence rate and case fatality rate among LTCF residents and care workers in facilities.

## Introduction

The outbreak of the severe acute respiratory syndrome coronavirus 2 (SARS-CoV-2) infection led to a pandemic, and it has become apparent that the virus disproportionately affects older individuals and those with more comorbidities, among whom mortality is the highest. Mortality due to coronavirus disease (COVID-19) among elderly individuals aged 65 years or older is high compared to other age groups [1]. Residents of long-term care facilities (LTCFs) are particularly susceptible. The mortality rate of residents in LTCFs owing to COVID-19 was reported to be higher than that of community-dwelling older persons (age 65 years or older) [2].

Regarding the risk of contracting a SARS-CoV-2 infection, residents in LTCFs harbour a disproportionately high risk compared to elderly people of the same age living at home, owing to a greater likelihood of individuals exposed to asymptomatic and pre-symptomatic care workers, family members and outsourcers, and a higher potential risk for aerosol exposure through sputum suction or from ventilated residents [3]. LTCFs also afford a high possibility of progression to a massive outbreak owing to the congregate living environments. The capacities of LTCFs for emergency management are limited [4]. In addition, the shortage of care workers at LTCFs has been a common concern in most facilities, even before the COVID-19 outbreak [5–7]. In the event of an outbreak in such LTCFs, in addition to the vulnerable population and congregate living environments, the management of resources becomes challenging because infections among care workers result in escalating shortages of personnel. Difficulties in securing resources for testing and personal protective equipment hinder the early detection of infected individuals and the prevention of further transmission [1]. Decision-making skills in emergency settings may be limited but are critical for managing the situation within the facilities and for coordinating with external supporting partners such as local governments [7].

Under the COVID-19 response protocols of the Japanese Government, an outbreak is defined as the accumulation of reports of five or more SARS-CoV-2 polymerase chain reaction (PCR)-positive cases in one facility in a short period of time (generally 10 days), regardless of whether the cases are in residents or healthcare workers [8]. In the event of an outbreak at a LTCF, local public health centre personnel take histories and diagnose individual patients’ conditions, decide treatment settings, conduct contact tracing, examine environmental conditions, and advise patient management and measures to prevent the spread of infection [9]. However, when responsible public health centres are unable to manage the situation, the local government requests the assistance of the Ministry of Health, Labour and Welfare (MHLW). Upon receiving shared information about an outbreak, the MHLW deploys teams to the outbreak sites. For outbreaks at LTCFs, the Headquarters of the Disaster Medical Assistance Team (DMAT) deploy emergency response teams (ERTs) [10]. When outbreaks at LTCFs occur, the longer a situation of limited resources and the uncontrolled facility continues, the worse the incidence and case-fatality rates may be. The timeliness of ERTs starting their work onsite is considered to affect the outcomes of an outbreak.

### Objectives of the study

Therefore, the objectives of this study were to examine whether early-phase interventions by ERTs for COVID-19 outbreaks in LTCFs contribute to lower incidence and case fatality rates compared to LTCFs without ERT support and to analyse the required assistance by ERTs. The analyses were conducted considering the records of LTCFs and reports from ERTs that assisted in managing the outbreaks.

## Materials and methods

This retrospective cohort study was conducted to examine the convergence of outbreaks in LTCFs assisted by ERTs.

### LTCFs settings

There are three major settings for LTCFs in Japan: LTC hospitals, LTC nursing homes, and LTC homes. LTC hospitals are responsible for residents’ functional recovery via the provision of continuous medical treatment and rehabilitation for those who have received acute care. Treatment procedures such as gastrostomy, ventilatory support, and intravenous infusion are offered according to prescriptions by physicians [3]. Residents who require acute intensive medical care are transferred to other relevant hospitals. LTC nursing homes provide daily care to older adults with physical or mental disabilities who require intensive support in activities of daily living (such as bathing, dressing, and going to the bathroom) but not medical care in hospitals. Among them, 20–35% are bedridden [3]. LTC homes provide space to older adults with or without physical or mental disabilities. Even those with disabilities are relatively independent and can manage their daily lives. When residents require health consultations, they visit clinics outside the facilities.

### ERT settings

A guideline for the support activities of ERTs in response to the outbreak at LTCFs was approved by the MHLW at the end of April 2020. Therefore, organized support by ERTs to LTCFs started on May 1, 2020. The ERTs are trained in resource management, tactics integration, and collaborative actions in response to emergencies and infection control [11]. The training is based on an incident management system developed by the Federal Emergency Management Agency (FEMA) in the United States [12,13]. Professionals holding ERT qualifications are registered in advance with the MHLW and managed by pooled roster. When the MHLW receives a request for an ERT from a public health centre, a request is made simultaneously to pooled professionals, and the required number of ERTs are constituted and dispatched within 1–2 days of the request from the professionals available. The number of ERTs increased in response to the spread of infection and the pooled ERTs were able to respond to the requests for support to manage the outbreak without shortages or delays. The ERT provides supportive interventions according to its guidelines. They are applied regardless of the period of the study and the timing of intervention. There are eight principal major functions in the guideline: establishing command and coordination of the facility, consolidating the outbreak information, communication and information management, management of human and material resources, infection control, establishing a system to deliver medical and nursing care, livelihood support of residents, and mental care of staff. Each ERT dispatched to an LTCF outbreak analysed the current status of the facility, established a course of action, and implemented support in accordance with the eight principal major functions.

### Subjects

A total of 59 requests for support to manage outbreak incidents were made from LTCFs to MHLW throughout Japan between 1 May 2020 and 31 January 2021, from the dates ERTs support according to a guideline had become available and before the initiation of the nationwide COVID-19 vaccination. The requests from LTCFs were made through the local governments responsible for the management of incidents. ERTs were deployed to all 59 LTCFs and these 59 LTCFs were used as the subjects LTCFs in this study.

### Case identification

When multiple positive cases are detected and ERT is requested, information on all residents and care workers (nurses, nurse aids, and personal care helpers, whether full-time or part-time) who were on scheduled shifts to directly provide care to residents were listed. Then facility-wide PCR tests were performed for all listed residents and care workers. Health monitoring of the listed residents and care workers was performed by designated care workers (nurses or nurse aids trained for measuring vital signs) at the facilities in coordination with the advice of ERTs. During the health monitoring, when there was an onset of symptoms, PCR tests were performed according to the instructions by the public health centres. Health monitoring was performed from the onset of the index case to 14 days after the expiration of the infectable period of the last COVID-19-positive case. An “index case” is the very first case of an infectious disease that was identified in an outbreak. The onset of the index case was defined by the appearance of symptoms or COVID-19 PCR-positivity even without symptoms but routine surveillance testing was not performed in any of the subject LTCFs.

### Information filed

Records of residents and care workers and daily reports by ERTs were used for analysis. Items of information regarding (1) dates of ERT support operations, (2) residents, (3) care workers, and (4) daily reports by ERTs were filed. Information regarding dates of ERT support operations included the date of onset of the index case of the cluster, the date of confirmation of the index case of the cluster, the date an ERT started the support operation, the date 14 days after the end of the infectable period of the last case, and date an ERT finished the operation. Residents’ information included the number of residents in the report of the index case, age of the residents, diagnosis of COVID-19, and prognosis of the disease. Residents hospitalised for the treatment of COVID-19 were followed up, and their prognoses were reflected in the file. Information on care workers included the number of care workers on scheduled shifts at the report of the index case, diagnosis of COVID-19, and prognosis of the disease. Workers hospitalised for the treatment of COVID-19 were followed up, and their prognoses were reflected in the file. Information on residents and care workers were documented by persons responsible for the facilities and confirmed by ERTs. The finalised files were reported to the DMAT Secretariat headquarters. From these files, a dataset was extracted and used for the analysis. ERTs delivered daily reports of their observations, analyses, and operations at the facilities to the DMAT Secretariat headquarters. These reports were used for the analysis.

### Analysis

The number of incidents per 100 individuals and case-fatality rates (per 100 positive cases) among residents and care workers were calculated in the three types of LTCFs: hospitals, nursing homes, and homes. Baseline characteristics between early phase and late phase of ERT support were compared and tested using a log rank test. Rates in facilities supported by ERTs from the early or late phase of the onset of clusters were compared and tested using Fisher’s exact probability test. Early and late phase initiations of support were defined as situations where an ERT initiated support within 7 days and after 7 days from the date of confirmation of the index case, respectively. This classification was based on the infectivity of COVID-19 in the first and second waves, which was the strongest the first 3 days after onset, and the average incubation period was 4–5 days after exposure to the virus. Statistical analysis was performed using statistical software R (R Foundation, Vienna, Austria). Narrative daily reports of 59 ERTs regarding their support to LTCFs were reviewed to identify ERT activities at the LTCFs during the COVID-19 outbreak. Two independent researchers reviewed all narrative data and double-coded the ERT activities. Discrepancies in coding were resolved through continued review until both coders agreed. After identifying the types of activities based on the codes, functions according to the incident management protocols of FEMA were labelled [13]. The number of facilities where individual activities were provided by ERTs was determined for LTC hospitals, nursing homes, and homes.

### Ethics approval

Research ethics were reviewed and approved by the Institutional Review Committee of the Medical School of Tokyo Medical and Dental University (M2021-203). Personal identification information of residents and care workers as well as the identification information of facilities was excluded from the data set when provided by the DMAT Secretariat headquarters for analysis. Anonymised reports, without the names of the facilities, were used for the analysis of ERT activities at the LTCFs.

## Results

Table 1 shows the baseline characteristics of the LTCFs. Records of 6,432 residents (4,281 in hospitals, 1,395 in nursing homes, and 756 in homes) and 8,586 care workers (6,731 in hospitals, 1,289 in nursing homes, and 566 in homes) at 59 LTCFs supported by ERTs were filed. Among the facilities supported by ERTs, at 40 facilities, ERTs initiated their support in the early phase, and at 19 facilities, ERTs initiated their support in the late phase. The median number of days ERTs were deployed to individual facilities was 17·0, and the median number of days from the index case report to 14 days after the end of the infectable period of the last case was 33·2 days. The ERTs provided onsite support during the deployment period. After the withdrawal of the ERTs, the facilities were able to consult the teams remotely according to their needs. In all LTCFs supported by ERTs, LTC hospitals, LTC nursing homes, and LTC homes, except for the number of days between the index case report and initiation of support by ERTs, there were no significant differences in baseline characteristics between the early and late phase of initiation of support.

**Table 1:** Baseline characteristics of long-term care facilities (LTCFs).

		LTCFs supported by ERTs*^1^			
		All	Early phase (< 7 days)	Late phase (≥ 7 days)	*p* value*^2^
**LTCFs, All**	N, facilities	59	40	19	
Number of residents on report of the index case	N, residents	6432	4464	1968	
Number of care workers on the scheduled shift on report of the index case	N, care workers	8586	5225	3361	
*Indicators by facilities*					
Average age of residents	Median, years [Q1, Q3]	83·0 [75·1, 86·3]	82·5 [74·0, 86·1]	83·2 [77·1, 87·9]	0.365
Number of care workers per 100 residents	Average	133·5	117·0	170·8	0.328
Days between report of the index case and initiation of support by ERTs	Median [Q1, Q3]	4·0 [2·0, 7·0]	3·0 [2·0, 4·0]	9·0 [7·5, 13·5]	< 0·001
Days of support by ERTs	Median [Q1, Q3]	17·0 [9·5, 22·5]	18·0 [11·0, 23·0]	10·0 [5·5, 18·5]	0·057
Days from report of the index case to 14 days after the end of the infectable period of the last case	Average Median [Q1, Q3]	33·2 31·0 [20·5, 38·5]	31·3 27·0 [19·8, 37·3]	37·2 37·0 [22·5, 39·5]	0·200
**LTC Hospitals**	N, facilities	28	18	10	
Number of residents on report of the index case	N, residents	4281	2894	1387	
Number of care workers on the scheduled shift on report of the index case	N, care workers	6731	3753	2978	
*Indicators by facilities*					
Average age of residents	Median [Q1, Q3]	77·4 [69·0, 83·1]	76·0 [69·0, 81·4]	78·5 [73·0, 83·2]	0·286
Number of care workers per 100 residents	Average	157·2	129·7	214·7	0·724
Days between report of the index case and initiation of support by ERTs	Median [Q1, Q3]	5·0 [2·0, 7·3]	3·0 [2·0, 4·8]	9· 5 [7·3, 15·5]	< 0·001
Days of support by ERTs	Median [Q1, Q3]	19·5 [11·8, 25·5]	21·0 [15·0, 29·0]	17·5 [9·0, 22·0]	0·111
Days from report of the index case to 14 days after the end of the infectable period of the last case	Average Median [Q1, Q3]	38· 3 37·0 [26·8, 42·0]	36·3 35·0 [25·3, 42·0]	41·9 37·5 [36·3, 38·8]	0·280
**LTC Nursing Homes**	N, facilities	15	11	4	
Number of residents on report of the index case	N, residents	1395	1105	290	
Number of care workers on the scheduled shift on report of the index case	N, care workers	1289	1077	212	
*Indicators by facilities*					
Average age of residents	Median, years [Q1, Q3]	86·6 [81·0, 87·5]	85·8 [81·0, 86·7]	88·1 [77·4, 89·4]	0·22
Number of care workers per 100 residents	Average	92·4	97·5	73·1	0·08
Days between report of the index case and initiation of support by ERTs	Median [Q1, Q3]	3·0 [2·0, 7·0]	2·0 [1·5, 3·0]	11·5 [9·0, 26·3]	< 0·001
Days of support by ERTs	Median [Q1, Q3]	18·0 [12·0, 21·5]	19·0 [13·5, 21·5]	10·5 [2·5, 26·25]	0·47
Days from report of the index case to 14 days after the end of the infectable period of the last case	Average Median [Q1, Q3]	31·6 27·0 [18·5, 39·5]	28·7 27·0 [19·0, 36·5]	39·5 36·0 [17·8, 57·8]	0·78
**LTC Homes**	N, facilities	16	11	5	
Number of residents on report of the index case	N, residents	756	465	291	
Number of care workers on the scheduled shift on report of the index case	N, care workers	566	395	171	
*Indicators by facilities*					
Average age of residents	Median, years [Q1, Q3]	85·5 [82·7, 86·4]	86·0 [83·9, 86·2]	84·0 [80·0, 88·7]	0·98
Number of care workers per 100 residents	Average	74·9	84·9	58·8	0·17
Days between report of the index case and initiation of support by ERTs	Median [Q1, Q3]	4·5 [2·8, 7·0]	3·0 [2·0, 4·5]	8·0 [7·0, 12·0]	< 0·001
Days of support by ERTs	Median [Q1, Q3]	9·5 [4·8, 13·3]	11·0 [3·5, 17·5]	9·0 [6·0, 9·0]	0·46
Days from report of the index case to 14 days after the end of the infectable period of the last case	Average Median [Q1, Q3]	25·6 21·5 [19·0, 28·8]	25·7 20·0 [18·5, 29·0]	25·8 22·0 [21·0, 27·0]	0·49

(*1) Emergency response teams.

(*2) *P*-value comparing values for the early-phase and late-phase intervention groups, by log-rank test.

Report of LTCFs supported by ERTs from 1st May 2020 to 31st January 2021.

Table 2 shows COVID-19 incidence and case-fatality rates among residents at the LTCFs. Overall incidence and case-fatality rates of residents in LTCFs supported by ERTs were 34·3% and 15·5%, respectively. Among all types of LTCFs with ERT support, the incidence rate in LTC nursing homes (39·5%) was significantly higher than that in LTC hospitals (31·2%) [OR  =  1·44; 95% CI (1·27, 1·63)], and the incidence rate in LTC homes (24·3%) was significantly lower than that in LTC hospitals [OR  =  0·69: 95% CI (0·57, 0·83)]. The case-fatality rate in LTC nursing homes (10·9%) was significantly lower than that in LTC hospitals (18·4%), [OR  =  0·51; 95% CI (0·37, 0·69)], and the case-fatality rate in LTC homes (8·7%) was significantly lower than that in LTC hospitals [OR  =  0·71; 95% CI (0·59, 0·85)]. The results of comparing incidence rates by phase of intervention showed significantly lower incidence rates in facilities of early-phase intervention than in facilities of late-phase intervention among all LTCFs (p < 0·001), LTC hospitals (p < 0·001), LTC nursing homes (p  =  0·031), and LTC homes (p  =  0·015).

**Table 2:** COVID-19 incidence and case-fatality rates among residents at long-term care facilities (LTCFs).

	LTCFs supported by ERTs*^1^			
	All	Early-phase intervention (< 7 days)	Late-phase intervention (≥ 7 days)	*p* value*^2^
**LTCFs, All**				
Number of residents on report of the index case	6038	4464	1574	
Number of residents confirmed positive for SARS-CoV-2	2071	1351	720	
Number of deaths among positive cases	322	200	122	
Incidence rate (positive cases per 100)	34·3	30·3	36·6	< 0·001
Case-fatality rate (per 100 positive cases)	15·5	14·8	16·9	0·203
**LTC Hospitals**				
Number of residents on report of the index case	4281	2894	1387	
Number of residents confirmed positive for SARS-CoV-2	1336	832	504	
Number of deaths among positive cases	246	148	98	
Incidence rate (positive cases per 100)	31·2	28·7	36·3	< 0·001
Case-fatality rate (per 100 positive cases)	18·4	17·8	19·4	0·467
**LTC Nursing Homes**				
Number of residents on report of the index case	1395	1105	290	
Number of residents confirmed positive for SARS-CoV-2	551	420	131	
Number of deaths among positive cases	60	42	18	
Incidence rate (positive cases per 100)	39·5	38·0	45·2	0·031
Case-fatality rate (per 100 positive cases)	10·9	10·0	13·7	0·260
**LTC Homes**				
Number of residents on report of the index case	756	465	291	
Number of residents confirmed positive for SARS-CoV-2	184	99	85	
Number of deaths among positive cases	16	10	6	
Incidence rate (positive cases per 100)	24·3	21·3	29·2	0·015
Case-fatality rate (per 100 positive cases)	8·7	10·1	7·1	0·602

(*1) Emergency response teams.

(*2) *P*-value comparing values for the early-phase and late-phase intervention groups, by Fisher test.

Report of LTCFs supported by ERTs from 1st May 2020 to 31st January 2021.

Table 3 shows COVID-19 incidence rates among care workers at LTCFs. There was no fatal case among the care workers. The incidence rate among care workers in all LTCFs supported by ERTs was 11·5%. The incidence rate in LTC nursing homes (21·1%) was significantly higher than that in LTC hospitals (9·4%) [OR  =  2·57; 95% CI (2·12, 3·02)]. The results of comparing incidence rates by phase of intervention showed significantly lower incidence rates in facilities with early-phase interventions than in those with late-phase interventions among all LTCFs (p  = 0·011), LTC hospitals (p < 0·001), and LTC homes (p  =  0·008).

**Table 3:** COVID-19 incidence rate among care workers at long-term care facilities (LTCFs).

	LTCFs supported by ERTs*^1^			
	All	Early-phase intervention (< 7 days)	Late-phase intervention (≥ 7 days)	*p* value*^2^
**LTCFs, All**				
Number of care workers on report of the index case	8586	5225	3361	
Number of care workers confirmed positive for SARS-CoV-2	984	562	422	
Number of deaths among positive cases	0	0	0	
Incidence rate (positive cases per 100)	11·5	10·8	12·6	0·011
**LTC Hospitals**				
Number of care workers on report of the index case	6731	3753	2978	
Number of care workers confirmed positive for SARS-CoV-2	634	294	340	
Number of deaths among positive cases	0	0	0	
Incidence rate (positive cases per 100)	9·4	7·8	11·4	< 0·001
**LTC Nursing Homes**				
Number of care workers on report of the index case	1289	1077	212	
Number of care workers confirmed positive for SARS-CoV-2	272	224	48	
Number of deaths among positive cases	0	0	0	
Incidence rate (positive cases per 100)	21·1	20·8	22·6	0·581
**LTC Homes**				
Number of care workers on report of the index case	566	395	171	
Number of care workers confirmed positive for SARS-CoV-2	78	44	34	
Number of deaths among positive cases	0	0	0	
Incidence rate (positive cases per 100)	13·8	11·1	19·9	0·008

(*1) Emergency response teams.

(*2) *P*-value comparing values for the early-phase and late-phase intervention groups, by Fisher test.

Report of LTCFs supported by ERTs from 1st May 2020 to 31st January 2021.

Table 4 shows the results of the 18 identified activities of ERTs during the COVID-19 outbreak at LTCFs, correspondence with the principal major functions in the guideline, and the number of LTCFs that received support from ERTs. Support activities related to command and coordination, consolidating the outbreak information, and communication and information management were required at most facilities. Concerning decision-making, all facilities required support from ERTs. A total of 42 facilities required assistance and supervision for infection control. Additionally, in terms of resource management, the proportions of facilities that required support for personnel management among LTC hospitals and LTC nursing homes were 64·3% and 73·3%, respectively. Assistance with general operational functions of facilities, including meals, laundry, garbage disposal, and cleaning, was required at 67·9% of the LTC hospitals. Assistance with an improvement of the facility’s work environment, including caring for the mental health of the staff, was also required in 67·9% of the LTC hospitals.

**Table 4:** Activities by emergency response teams (ERTs) during the COVID-19 outbreak in long-term care facilities (LTCFs) and the correspondence with the principal major functions of ERTs.

Activities by ERTs during the COVID-19 outbreak	Principal major functions of ERTs	LTC Hospitals (N = 28)		LTC Nursing Homes (N = 15)		LTC Homes (N = 16)	
		n	%	n	%	n	%
Analysis of staff shortages, including estimates of when staff members on sick leave can return and recruitment of new staff members	Personnel resource management	18	64·3	11	73·3	5	31·3
Assistance in requests to local governments for oxygen concentrators	Material resource management	0	0	4	26·7	5	31·3
Analysis of stock and use of personal protective equipment and assistance in requests to local governments for more	Material resource management	15	53·6	5	33·3	5	31·3
Assistance in recruitment of temporary medical staff members and coordination of staffing	Personnel resource management	18	64·3	11	73·3	8	50·0
Securement of space for an emergency response operation centre in the facility and creation of isolation areas for patients	Material resource management	7	25·0	4	26·7	2	12·5
Procurement of transportation for patient transfer	Material resource management	11	39·3	8	53·3	10	62·5
Assessment of facility’s overall situation	Consolidating outbreak information	23	82·1	15	100·0	14	87·5
Analysis of overall status of outbreak and related conditions in the facility	Consolidating outbreak information	27	96·4	15	100·0	16	100·0
Assistance in establishment of decision-making approaches and development of response plans	Command and Coordination	28	100·0	15	100·0	16	100·0
Exchange of information with front-line staff and assistance in regular meetings devoted to them	Communication and Information management	20	71·4	15	100·0	16	100·0
Communication with public health centres, local governments, and other institutions and development of contact lists containing them	Communication and Information management	26	92·9	15	100·0	16	100·0
Dissemination of information to institution’s staff members	Communication and Information management	11	39·3	7	46·7	8	50·0
Dissemination of information to residents and their families	Communication and Information management	1	3·6	3	20·0	2	12·5
Dissemination of information to the media and general public	Communication and Information management	22	78·6	15	100·0	15	93·8
Assistance in and supervision for infection control such as guidance on zoning, separation of traffic lines between staff members, thorough wearing and removal of personal protective equipment	Infection control	21	75·0	9	60·0	12	75·0
Assistance in monitoring health conditions of residents, staff members, external personnel, and visitors	Establishing a system to deliver medical and nursing care	11	39·3	7	46·7	5	31·3
Assistance in general operational functions, including meals, laundry, garbage disposal, and cleaning	Livelihood support of residents	19	67·9	3	20·0	6	37·5
Assistance in improvement of the facility’s work environment, including the mental health of staff members	Mental care of staff	19	67·9	7	46·7	8	50·0

## Discussion

This retrospective cohort study analysed the data of 6,432 residents and 8,586 care workers at 59 LTCFs that received ERT assistance for the management of COVID-19 outbreak between May 2020 and January 2021 in Japan. A total of 2,071 and 984 PCR-positive cases among residents and care workers, respectively, were identified. Fatality associated with COVID-19 was reported for 322 residents but no care workers. Incidence and case fatality rates among residents and incidence rates among care workers were significantly lower in facilities supported by the early-phase intervention than in those by the late-phase intervention. Content analysis of activities by ERTs showed that LTCF outbreaks required support that was not limited to assistance and supervision in infection control but also included assistance with command and coordination, resource management, and general operational and other functions in the facilities.

A systematic review of 49 reports related to COVID-19 outbreaks in LTCFs from 14 of the Organisation for Economic Co-operation and Development (OECD) countries reported that the pooled incidence and case-fatality rates of residents were 45% [95% confidence interval (CI) 32–58%] and 23% [95% CI 18–28%], respectively, between December 2019 and September 2020 [14]. The overall incidence rate (34·3%) and case-fatality rate (15·5%) of residents in the current study of LTCFs in Japan supported by ERTs according to the outbreaks were slightly lower than the pooled averages reported in the review. However, the incidence rate in LTC nursing homes was 39·5%, and the case-fatality rate in LTC hospitals was 18·4%. These figures are closer to the pooled averages for LTCFs reported in the review report. The population-wide incidence rate of COVID-19 in Japan for those aged ≥ 70 years by January 2021 was 207·7 per 100,000 population. This is far lower than that in other OECD countries, where the rate is reported to be over 8,000 per 100,000 people [14]. Despite the relatively low incidence rate for the older population in Japan in general, both incidence and case-fatality rates among residents in LTCFs that experienced outbreaks were similar to those in other OECD countries. It is noteworthy, once outbreaks occurred in LTCFs, the incidence and case-fatality rates among residents within the facilities were similarly high as in LTCFs in other countries, although the population-wide incidence rates were relatively lower than that of other countries. Strategies to prevent outbreaks in LTCFs should be prioritised.

Three factors are considered to explain the significantly lower incidence rates in LTC homes than other facilities. First, there was a difference in the use of private rooms by residents. While residents in LTC homes reside in private isolated rooms avoiding close contact with other residents, most residents in LTC hospitals and nursing homes stay in multi-bed rooms [15]. Second, residents in LTC homes are relatively healthier than residents in LTC nursing homes and hospitals because only residents who do not require sustained medical intervention can reside in LTC homes. Third, residents in LTC homes are relatively more independent with a lower requirement of care workers leading to lower chances of spread of the disease from care workers to residents [4]. Thus, the management of outbreaks in LTC hospitals and nursing homes faces difficulties because of limited availability of isolated rooms, the requirement of medical interventions to the residents, and the requirement of nursing care with close contact with the residents. The overall case-fatality rate was at its highest in LTC hospitals, followed by LTC nursing homes and LTC homes. This finding may be attributed to the fact that a larger proportion of residents in LTC hospitals have underlying conditions associated with COVID-19 severity, such as cerebrovascular, cardiovascular and diabetes, and many residents of LTC hospitals are also being treated with medical procedures such as gastrostomy, ventilatory support, or intravenous infusion leading to poor original immune status [3]. Once infected with SARS-CoV-2, the prognosis for these residents is generally poor.

Regarding occurrence among care workers, the total incidence rate among care workers was the highest in LTC nursing homes, followed by LTC homes and hospitals. Two factors were considered to explain these results. First, there is the availability of a fewer number of care workers in LTC nursing homes than LTC hospitals [3]. Upon the onset of an outbreak, individual workers are faced with an increasing burden of duties. Facilities with reasonable numbers of staff members have the advantage of managing infections among care workers. Second, care workers in LTC nursing homes may have been less familiar with infection control measures during an outbreak than those in LTC hospitals [16]. Care workers without proper infection control education are at a significant risk of transmitting the virus to both residents and other care workers. Among care workers, deaths were not reported. This was interpreted because care workers were younger and had better functions in daily life than residents. Compared to LTC hospitals and LTC homes, LTC nursing homes have more residents who can communicate but need assistance with all daily activities, and therefore, exposure to infection from prolonged intense contact with residents is unavoidable. These characteristics may have influenced the fact that the early phase support made no significant difference in the incidence rate of LTC nursing home healthcare workers [3].

The results of the content analysis showed that many facilities required support for infection control. However, in addition to infection control, there was resource and personnel management to consider, and support related to residents’ day-to-day lives was also necessary [17]. The support provided by ERTs was in line with resource management, command and coordination, and communication and information management. More than 90% of all LTCFs needed assistance related to command and coordination, which is fundamental for the operational governance of the facilities. The members of ERTs in Japan had experience in providing such support to medical institutions during natural disasters and were able to transfer expertise to the management of out-of-control situations encountered in LTCF outbreak emergencies.

Upon the occurrence of outbreaks in LTCFs, care workers generally experience the fear of contracting the infection, and the provision of the usual management of care services to residents becomes difficult [18]. Even communication with local governments and partner institutions does not function well [18]. The advantages of early-phase intervention by ERTs are believed to strengthen infection control and reorganise the functions of facilities by the proper capacity deployment of workers and procurement of equipment and sanitary materials. The early-phase intervention also helps establish command and control functions to manage the spread of infection in the facility. Early-phase intervention helps minimise the uncontrolled period when compared with late-phase intervention. On the other hand, for the facilities helped by ERTs from the late-phase, public health centres responsible for the facilities took longer times to grasp the critical situation and a need for ERTs [19].

Contingency planning includes preparation for accurate and prompt information reporting and communication, promoting leadership by creating an organisational chart and establishing decision-making approaches, recruiting human resources, and ensuring occupational health and resource availability [20]. Formulating such planning before the incident is required to mitigate the damage [21]. Some studies have shown that contingency plan strategies help improve readiness, allow effective use of resources, reduce the spread of the disease, and increase the acceptability of emergency interventions [21]. The support provided by ERTs in LTCFs during outbreaks has some features in common with contingency planning. If the latter can be established before an outbreak, there is a likelihood that the functions of the facilities will be maintained without the assistance of ERTs, making it an important consideration in preparedness for infectious disease outbreaks.

A notable strength of this study is the use of a large sample of data collected from all LTCFs supported by ERTs throughout the country. The number of all outbreaks of LTCFs during the observation period, including small outbreaks that were responded by public health centres, was 729 [22]. Of these, data were obtained from all 59 facilities where it was difficult for public health centres to respond and a request for assistance was made to the MHLW. In that context, the results represent the LTCFs with outbreaks in Japan that required assistance from other than public health centres in general.

One of the limitations is that this study is a comparison of early-phase intervention and late-phase intervention and did not include the information without ERT intervention. Potential factors that might influence incidence and fatality rates, such as weather conditions, seasons, the built-in environmental structure of the facility, and responses by local public health centres, were not considered in the analysis. Further studies should address these factors to elucidate the effectiveness of ERT participation. The fact that the Japanese healthcare system differs from the healthcare systems of other countries can be another limitation. However, in countries that have a small and highly mobile ERT system that operates in the event of natural disasters, as is the case in Japan, it is possible to expect similar effects by implementing the same approach for LTCFs in the event of an outbreak.

This study examined the value of intervention by ERTs when incidents of COVID-19 infection arose in LTCFs throughout Japan. Compared to late-phase interventions, the results demonstrated a significant decline in incidence rate and case fatality rate among LTCF residents and care workers in facilities receiving early-phase assistance. The effective assistance from ERT intervention was not limited to infection control but extended to critical aspects of operational management, including command and control functions, resource management, and communication and information management, suggesting the importance of assistance in organizational governance in response to outbreaks at facilities.

## Supporting information

S1 File (TXT)

S2 File (TXT)
